# Temporal and Geographical Variability of Prevalence and Incidence of Autism Spectrum Disorder Diagnoses in Children in Catalonia, Spain

**DOI:** 10.1002/aur.2172

**Published:** 2019-07-17

**Authors:** Laura Pérez‐Crespo, Albert Prats‐Uribe, Aurelio Tobias, Enric Duran‐Tauleria, Ricard Coronado, Amaia Hervás, Mònica Guxens

**Affiliations:** ^1^ ISGlobal Barcelona Spain; ^2^ Pompeu Fabra University Barcelona Spain; ^3^ Spanish Consortium for Research on Epidemiology and Public Health (CIBERESP) Instituto de Salud Carlos III Madrid Spain; ^4^ Preventive Medicine and Public Health Training Unit, Parc de Salut Mar—Pompeu Fabra University—Public Health Agency of Barcelona Barcelona Spain; ^5^ Centre for Statistics in Medicine, Botnar Research Centre, NDORMS University of Oxford, Oxford UK; ^6^ Institute of Environmental Assessment and Water Research (IDAEA) Spanish Council for Scientific Research (CSIC) Barcelona Spain; ^7^ Institut Global d'Atenció Integral del Neurodesenvolupament (IGAIN) Barcelona, Spain; ^8^ Hospital General de Granollers Granollers, Spain; ^9^ Department of Pediatrics, Obstetrics and Gynecology and Preventive Medicine and Public Health, Autonomous University of Barcelona, Barcelona, Spain; ^10^ Child and Adolescent Mental Health Unit Hospital Universitari Mútua de Terrassa Barcelona Spain; ^11^ Department of Child and Adolescent Psychiatry/Psychology Erasmus University Medical Centre–Sophia Children's Hospital Rotterdam The Netherlands

**Keywords:** autistic disorder, child development disorder, neurodevelopmental disorders, epidemiology, Europe

## Abstract

This study aims to estimate the prevalence of autism spectrum disorders (ASD) in 2017 and the ASD diagnosis incidence between 2009 and 2017 in children living in Catalonia region in Spain, and their temporal and geographical variability. We used administrative data for all children aged 2–17 years who were insured in the public Catalan Health System between 2009 and 2017. We identified all ASD cases diagnosed between 2009 and 2017 (ICD‐9 codes 299.0, 299.1, 299.8, and 299.9). We estimated the ASD prevalence in 2017 and the overall annual incidence between 2009 and 2017, then stratified by sex, age group, and healthcare area. We used Poisson regression models to assess temporal trends in the incidence and mixed‐effects Poisson regression models to assess geographical variability. We observed an ASD prevalence of 1.23% (95% confidence interval [CI] 1.21–1.25) in 2017, with 1.95% (95% CI 1.92–1.99) for boys and 0.46% (95% CI 0.44–0.48) for girls, the highest prevalence being in 11‐ to 17‐year‐olds (1.80%, 95% CI 1.76–1.83). The ASD diagnosis incidence increased from 0.07% (95% CI 0.06–0.09) in 2009 to 0.23% (95% CI 0.21–0.24) in 2017, with a higher increase in girls, and in children aged 2–5 years at the time of diagnosis. We only observed geographical differences in prevalence in the 2017 data. We also detected a threefold increase in the diagnosis incidence overall, which was even more pronounced in girls and at early ages. In conclusion, the ASD prevalence observed in this study was 1.23% in 2017, with a sex ratio of 4.5 in favor of boys, which is consistent with previous studies. ***Autism Res*** *2019*. © 2019 International Society for Autism Research, Wiley Periodicals, Inc.

**Lay Summary:**

Autism spectrum disorders (ASD) are currently well known in our society as one of the most common neurodevelopmental disorders during childhood. The results of our study showed that, in 2017 in Catalonia, slightly more than one in a 100 children had an ASD diagnosis, it was more common in boys than in girls, and also in older children. In addition, between 2009 and 2017, we observed an increase in the number of new cases diagnosed each year. The data presented in this study will assist in planning and evaluating the needs of health services in this geographical region.

## Introduction

Autism spectrum disorders (ASD) are a group of neurodevelopmental conditions caused by atypical brain development that begins during prenatal or early postnatal life [Zachor & Curatolo, 2014]. ASDs are commonly characterized by impaired social interaction, deficits in social communication, and restricted, repetitive, and stereotyped behavior patterns [American Psychiatric Association, 2013]. The symptoms are clinically heterogeneous, and some patients are able to have an independent and fulfilling life with little support, while others have a more severe form that interferes with their quality of life [Farley et al., 2009; Hervas, 2016]. Additionally, individuals with ASD have a very high prevalence of comorbidities, which could explain the complex, and still not fully understood, etiology of the disorder [Murray et al., 2014]. Although considerable effort has been devoted to the etiological factors of ASD, no single cause has been identified. The available data suggest that this condition results from different sets of causal factors, including genetic and environmental risk factors [Huguet, Ey, & Bourgeron, 2013; Modabbernia, Velthorst, & Reichenberg, 2017].

ASD are a major public health problem due to the worldwide increase in their prevalence in recent decades reaching values of between 0.01% in Oman [Al‐Farsi et al., 2011] and 2.64% in South Korea [Kim et al., 2011]. The observed differences between studies might be due to methodological, cultural, and environmental factors [Hansen, Schendel, & Parner, 2015; Lyall et al., 2017]. Therefore, it is difficult to compare estimates of ASD prevalence and incidence from different studies because they may not have been conducted homogeneously. ASD epidemiological studies have mainly been based on registers or on multistage screening approaches. Most studies have been performed in America and Europe, whereas less data is available for the Western Pacific, South East Asia, and Eastern Mediterranean regions [Chaaya, Saab, Maalouf, & Boustany, 2016; Chinawa et al., 2017; Davidovitch, Hemo, Manning‐Courtney, & Fombonne, 2013; Dekkers, Groot, Díaz Mosquera, Andrade Zúñiga, & Delfos, 2015; Elsabbagh et al., 2012; Fombonne, 2009b, 2009a; Fortea Sevilla, Escandell Bermúdez, & Castro Sánchez, 2013; Hewitt et al., 2016; Hill, Zuckerman, & Fombonne, 2014; Huang et al., 2014; Idring et al., 2015; Isaksen, Diseth, Schjølberg, & Skjeldal, 2013; May, Sciberras, Brignell, & Williams, 2017; Pelly, Vardy, Fernandez, Newhook, & Chafe, 2015; Poovathinal et al., 2016; Raina et al., 2017; Rudra et al., 2017; Saemundsen, Magnússon, Georgsdóttir, Egilsson, & Rafnsson, 2013; Skonieczna‐Żydecka, Gorzkowska, Pierzak‐Sominka, & Adler, 2017; Sun et al., 2015; van Bakel et al., 2015; Zahorodny et al., 2014]. Studies carried out during the last two decades indicate that North America and Europe have the highest prevalences of ASD, with median rates of 0.90% and 0.61%, respectively. In the Western Pacific, ASD prevalence estimates are slightly lower, with a median rate of 0.50%, while in South America, Eastern Mediterranean, and South East Asia, ASD prevalence estimates were much lower, with median rates not exceeding 0.39%. In Europe, several studies have been conducted, mostly in Northern countries, and have used existing registers. However, in Spain, the most recent studies of ASD were based on a two‐phase screening approach. The first study that used this approach was conducted in the Canary Islands in 2012, and reported an estimated ASD prevalence of 0.61% in toddlers [Fortea Sevilla et al., 2013]. The other study was conducted in one area of Catalonia and showed an estimated ASD prevalence of 1.55% in preschool children, and 1.00% in primary school children [Morales‐Hidalgo, Roigé‐Castellví, Hernández‐Martínez, Voltas, & Canals, 2018]. Regarding sex differences in ASD diagnosis, most previous studies have shown a higher ASD prevalence in boys than in girls, with a median sex ratio of 4 (boy:girl) [Elsabbagh et al., 2012]. Although ASD symptoms tend to manifest themselves during early childhood, [American Psychiatric Association, 2013] some studies have reported higher ASD prevalence in children aged 4–9 years [Al‐Farsi et al., 2011; Skonieczna‐Żydecka et al., 2017; Yeargin‐Allsopp et al., 2003]. Furthermore, although few studies have assessed ASD incidence, they have all shown an increased incidence in the last years [Barbaresi, Katusic, Colligan, Weaver, & Jacobsen, 2005; Chien, Lin, Chou, & Chou, 2011; Davidovitch et al., 2013; Hinkka‐Yli‐Salomäki et al., 2014; Honda, Shimizu, Misumi, Niimi, & Ohashi, 1996; Jensen, Steinhausen, & Lauritsen, 2014; Lauritsen, Pedersen, & Mortensen, 2004; Nassar et al., 2009; Pelly et al., 2015; Plubrukarn, Piyasil, Moungnoi, Tanprasert, & Chutchawalitsakul, 2005; Powell et al., 2000; Raz, Weisskopf, Davidovitch, Pinto, & Levine, 2015; Taylor, Jick, & MacLaughlin, 2013; Williams et al., 2005; Wong & Hui, 2008]. As for prevalence, the incidence varies notably between countries and regions; the incidence in Europe and North America is approximately 0.15% [Davidovitch et al., 2013; Diallo et al., 2018; Jensen et al., 2014; Pelly et al., 2015; Raz et al., 2015; Taylor et al., 2013], while that in South Asia and Western Pacific regions is ~0.05% [Chien et al., 2011; Plubrukarn et al., 2005; Williams et al., 2005; Wong & Hui, 2008].

Overall, very few studies have been carried out in Southern European countries, particularly in our region, where little is known about the age of diagnosis and the distribution of prevalence and incidence of diagnosis in each sex. These data are crucial to understanding how ASD diagnosis is evolving, and to evaluating and planning the needs of the healthcare system. Therefore, the aim of this epidemiological study was to assess the prevalence of ASD in 2017, and its incidence of diagnosis between 2009 and 2017 among the child population of the Catalonia region in Spain, as well as their temporal and geographical variability.

## Methods

### *Population and Study Design*

We used administrative data for children insured under the Catalan Public Health Service between 2009 and 2017. In this cohort, we identified children diagnosed with ASD at public healthcare centers between 2009 and 2017, and we defined population‐based denominators based on the total population of children in 2017, and the total population of children at risk for each year between 2009 and 2017 (described below). Spain's National Health System provides universal access to health services for all native and foreign‐born children [García‐Armesto, Begoña Abadía‐Taira, Durán, Hernández‐Quevedo, & Bernal‐Delgado, 2010], regardless of socioeconomic/financial status or racial/ethnic identification [García‐Altés, Ruiz‐Muñoz, Colls, Mias, & Martín Bassols, 2018; Rajmil, 2000]. This study was reviewed and approved by the Clinical Research Ethics Committee of the Parc de Salut Mar.

### *ASD Cases*

In Catalonia, all children diagnosed with ASD in a public healthcare center are registered by the Catalan Health Service in the Minimum Basic Data Set register. This population register collects information on the patients' activity and health morbidity, and is populated using information provided by four different types of public healthcare centers: mental health centers, mental health outpatient clinics, primary care centers, and hospital discharges.

In this study, we included 2‐ to 17‐year‐old children with ASD diagnosed between 2009 and 2017 based on the following International Classification of Diseases 9^th^ edition (ICD‐9) codes: 299.0 (autistic disorder), 299.1 (childhood disintegrative disorder), 299.8 (other specified pervasive developmental disorders), and 299.9 (unspecified pervasive developmental disorder). In the register, ASD cases might be duplicated when they are registered in different public healthcare centers or by different health professionals. For our study, we selected the first entry in the register as the first ASD diagnosis as children have a unique identifier in all centers. We excluded ASD cases registered before the age of 2 because ASD diagnosis before this age might not be reliable [American Psychiatric Association, 2013; Charman & Baird, 2002].

The Catalan territory is divided into 29 healthcare areas based on geographic, socio‐economic, and demographic factors. For each patient, we collected information about sex, healthcare area where ASD was diagnosed, and age (at diagnosis and in 2017). Age was stratified in three categories: 2–5 years, 6–10 years, and 11–17 years.

### *Population‐Based Denominators*

We estimated the total population of children in 2017 and the total population of children at risk for each year between 2009 and 2017, as registered by the Catalan Health Service through the Central Register of Insured Persons. The total population of children was defined as the number of 2‐ to 17‐year‐olds in 2017, and the total population of children at risk as the number of 2‐ to 17‐year‐olds who were ASD diagnosed‐free but still at risk of being diagnosed of ASD for each year between 2009 and 2017. For both population‐based denominators, we used the population on 1st of January of each year. Both population‐based denominators were stratified by sex, age group, and healthcare areas, using the same category groups as those for the ASD cases.

### *Statistical Analyses*

We calculated the ASD prevalence in 2017 by dividing the number of 2‐ to 17‐year‐olds with an ASD diagnosis in 2017 (numerator) by the total number of 2‐ to 17‐year‐olds in the population in 2017(denominator). The ASD prevalence in 2017 was stratified by sex, age group (age in 2017), and healthcare area. The differences in ASD prevalence between sexes, age groups, and healthcare areas were estimated using the chi‐squared test. We created a map of the distribution of ASD prevalence by healthcare area.

We calculated the annual ASD diagnosis incidence between 2009 and 2017 by dividing the number of newly diagnosed cases of ASD among 2‐ to 17‐year‐olds (numerator) by the total population at‐risk 2‐ to 17‐year‐olds for each year (denominator). Annual ASD diagnosis incidence between 2009 and 2017 was stratified by sex, age group (at diagnosis), and healthcare area. As observations were independent between years, we used Poisson regression models to assess the annual trend of the overall ASD diagnosis incidence between 2009 and 2017, and that for each sex and age group (at diagnosis). We performed mixed‐effects Poisson regression analysis to assess differences between healthcare areas according to sex and age group (at diagnosis). The Aran healthcare area was excluded because the mixed‐effects Poisson regression model did not converge for this area, most likely due to the low number of ASD cases diagnosed and the small population size. Finally, we also created maps of the distribution of ASD diagnosis incidence for each year and for each healthcare area. We provide the 95% confidence interval (95% CI) for all the variables analyzed.

Statistical analyses were performed using STATA (version 14.2; Stata Corporation, College Station, TX) and Open Epi (version 3.01; Dean AG, Sullivan KM, Soe MM), and the maps were drawn using QGIS (version 3.0.2 Girona; QGIS Development Team).

## Results

### *Characteristics of the Study Population*

In 2017, 1,326,666 children aged 2–17 years were insured under the Catalan Health Service. Boys (51.5%) and girls (48.5%) were almost equally represented, and with regards to age groups 2–5 years old were 22.7%, 6–10 years old 31.6%, and 11–17 years old 45.7%. The ratios were similar for the overall studied population and by sex and age groups for the other years of study. Out of the total studied population in 2017, 15,466 had been diagnosed with ASD. Most ASD cases were in the 6–10 years age group (48.2%) followed by the 2–5 years (30.3%), and the 11–17 years groups (21.5%).

### *ASD Prevalence in 2‐ to 17‐Year‐Olds in 2017*

**Figure 1:**
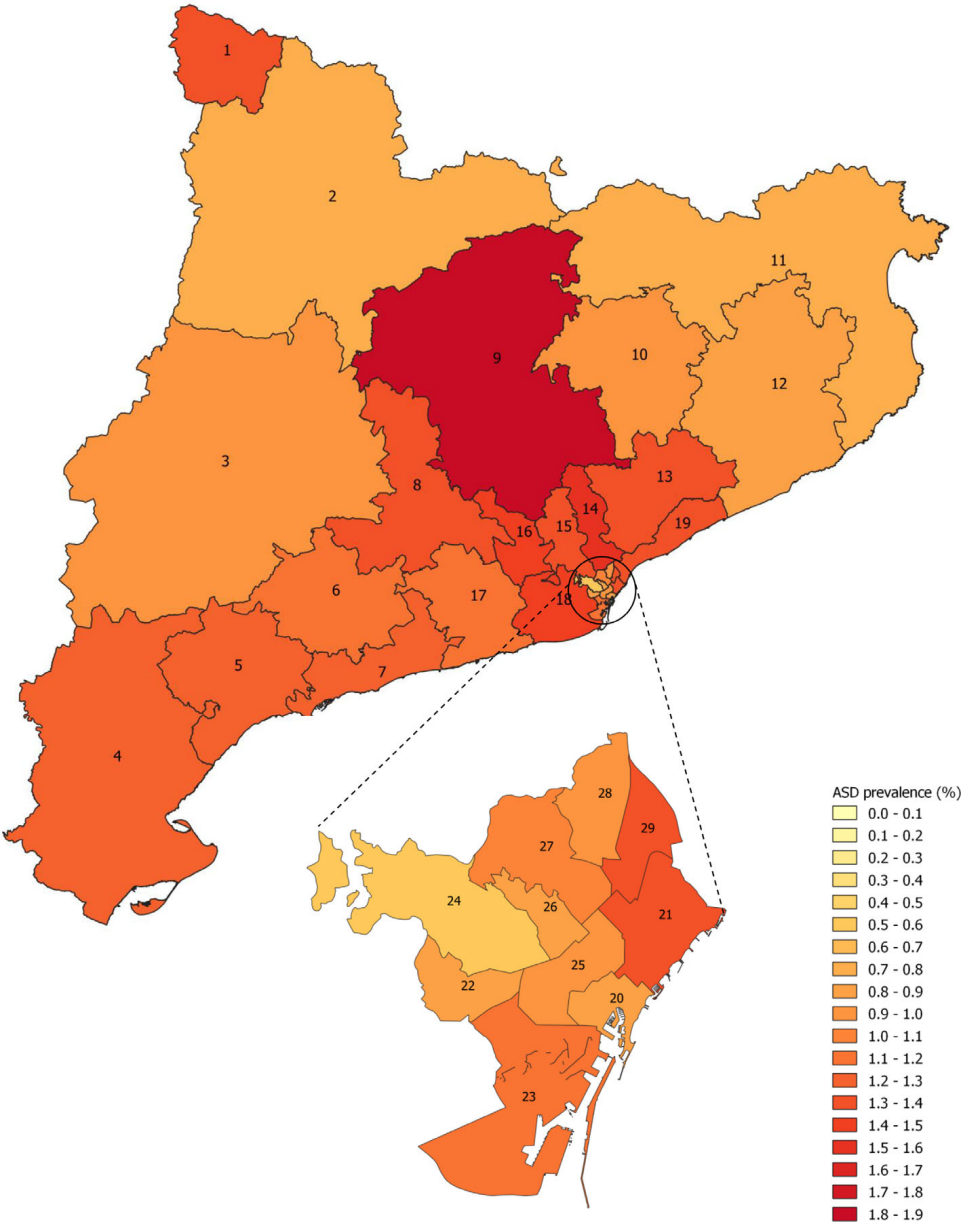
Autism spectrum disorders prevalence rate (%) among children from 2 to 17 years old in 2017 stratified by healthcare areas of Catalonia region, Spain. **1**: Aran; **2**: Alt Pirineu; **3**: Lleida; **4**: Terres de l'Ebre; **5**: Baix Camp–Priorat; **6**: Alt Camp–Conca de Barberà; **7**: Tarragonés–Baix Penedès; **8**: Anoia; **9**: Solsonès–Bages–Berguedà; **10**: Osona; **11**: Girona Nord; **12**: Girona Sud; **13**: Vallès Oriental; **14**: Vallès Occidental Est; **15**: Vallès Occidental Oest; **16**: Baix Llobregat Nord; **17**: Alt Penedès–Garraf; **18**: Baix Llobregat Centre‐Litoral i L'Hospitalet de Llobregat; **19**: Barcelonès Nord i Maresme; **20**: Barcelona Ciutat Vella; **21**: Barcelona Sant Martí; **22**: Barcelona les Corts; **23**: Barcelona Sants–Montjuïc; **24**: Barcelona Sarrià–Sant Gervasi; **25**: Barcelona Eixample; **26**: Barcelona Gràcia; **27**: Barcelona Horta–Guinardó; **28**: Barcelona Nou Barris; **29**: Barcelona Sant Andreu. Abbreviation: ASD, autism spectrum disorders.

We observed an overall ASD prevalence of 1.23% (95% CI 1.21–1.25) in 2017 (Table 1), significantly higher in boys (1.95%, 95% CI 1.92–1.99) than in girls (0.46%, 95% CI 0.44–0.48, *P* < 0.001) with 4.5‐fold higher prevalence in boys (12,647 boys vs. 2,819 girls). We also found significant differences in ASD prevalence between age groups according to the patient's age in 2017 (*P* < 0.001), with the highest prevalence in the 11–17 years age group (1.80%, 95% CI 1.76–1.83) followed by the 6–10 years (1.18%, 95% CI 1.14–1.21) and the 2–5 years groups (0.29%, 95% CI 0.26–0.32). Regarding geographical variability, the ASD prevalence varied significantly between healthcare areas in 2017 (*P* < 0.001), ranging from 0.55% (95% CI 0.46–0.64) in the Barcelona Sarrià‐Sant Gervasi healthcare area to 1.84% (95% CI 1.71–1.98) in Solsonès‐Bages‐Berguedà healthcare area (Fig. 1).

**Table 1:** Autism Spectrum Disorders Prevalence Rate (%) in 2017 and Diagnosis Incidence Rates (%) Between 2009 and 2017 by Sex and Age Group

			Sex			Age group^b^		
		Total	Boys	Girls	Sex‐ratio^a^	2–5 years old	6–10 years old	11–17 years old
***Prevalence***								
**2017**	*N*	15,466	12,647	2,819	4.5	869	4,937	9,660
	Prevalence rate (95% CI)	1.23 (1.21; 1.25)	1.95 (1.92; 1.99)	0.46 (0.44; 0.48)		0.29 (0.26; 0.32)	1.18 (1.14; 1.21)	1.80 (1.76; 1.83)
								
***Incidence***								
**2009**	*N*	919	768	151	5.1	168	390	361
	Incidence rate (95% CI)	0.07 (0.06; 0.09)	0.12 (0.10; 0.14)	0.02 (0.00; 0.05)		0.05 (0.02; 0.08)	0.10 (0.07; 0.13)	0.07 (0.05; 0.10)
**2010**	*N*	1198	1014	184	5.5	284	499	415
	Incidence rate (95% CI)	0.10 (0.08; 0.11)	0.16 (0.14; 0.18)	0.03 (0.01; 0.06)		0.08 (0.05; 0.11)	0.13 (0.10; 0.16)	0.08 (0.06; 0.11)
**2011**	*N*	1225	1004	221	4.5	282	548	395
	Incidence rate (95% CI)	0.10 (0.08; 0.11)	0.15 (0.13; 0.18)	0.04 (0.01; 0.06)		0.08 (0.05; 0.11)	0.14(0.11; 0.16)	0.08 (0.05; 0.10)
**2012**	*N*	1485	1238	247	5.0	398	635	452
	Incidence rate (95% CI)	0.12 (0.10; 0.13)	0.19 (0.17; 0.21)	0.04 (0.02; 0.07)		0.11 (0.08; 0.14)	0.16 (0.13; 0.18)	0.09 (0.06; 0.12)
**2013**	*N*	2029	1674	355	4.7	570	871	588
	Incidence rate (95% CI)	0.16 (0.14; 0.18)	0.26 (0.24; 0.28)	0.06 (0.03; 0.08)		0.16 (0.13; 0.19)	0.21 (0.18; 0.24)	0.12 (0.09; 0.14)
**2014**	*N*	2227	1791	436	4.1	541	961	725
	Incidence rate (95% CI)	0.18 (0.16; 0.19)	0.27 (0.25; 0.29)	0.07 (0.05; 0.10)		0.16 (0.13; 0.19)	0.23 (0.20; 0.26)	0.13 (0.10; 0.15)
**2015**	*N*	2393	1933	460	4.2	587	962	844
	Incidence rate (95% CI)	0.19 (0.17; 0.21)	0.30 (0.28; 0.32)	0.08 (0.05; 0.10)		0.18 (0.15; 0.21)	0.23 (0.20; 0.26)	0.14 (0.12; 0.17)
**2016**	*N*	2668	2144	524	4.1	695	1055	918
	Incidence rate (95% CI)	0.21 (0.20; 0.23)	0.33 (0.31; 0.35)	0.09 (0.06; 0.11)		0.22 (0.19; 0.25)	0.25 (0.22; 0.28)	0.18 (0.15; 0.20)
**2017**	*N*	2833	2272	561	4.1	756	1144	933
	Incidence rate (95% CI)	0.23 (0.21; 0.24)	0.35 (0.33; 0.37)	0.09 (0.07; 0.12)		0.25 (0.22; 0.28)	0.27 (0.25; 0.30)	0.17 (0.15; 0.20)

a Boys versus girls.

b Age groups based on child's age in 2017 for ASD prevalence and age groups at diagnosis for ASD incidence.

Abbreviations: CI, confidence interval; *N*, number of children with autism spectrum disorders.

### *ASD Diagnosis Incidence in 2‐ to 17‐Year‐Olds From 2009 to 2017*

**Figure 2:**
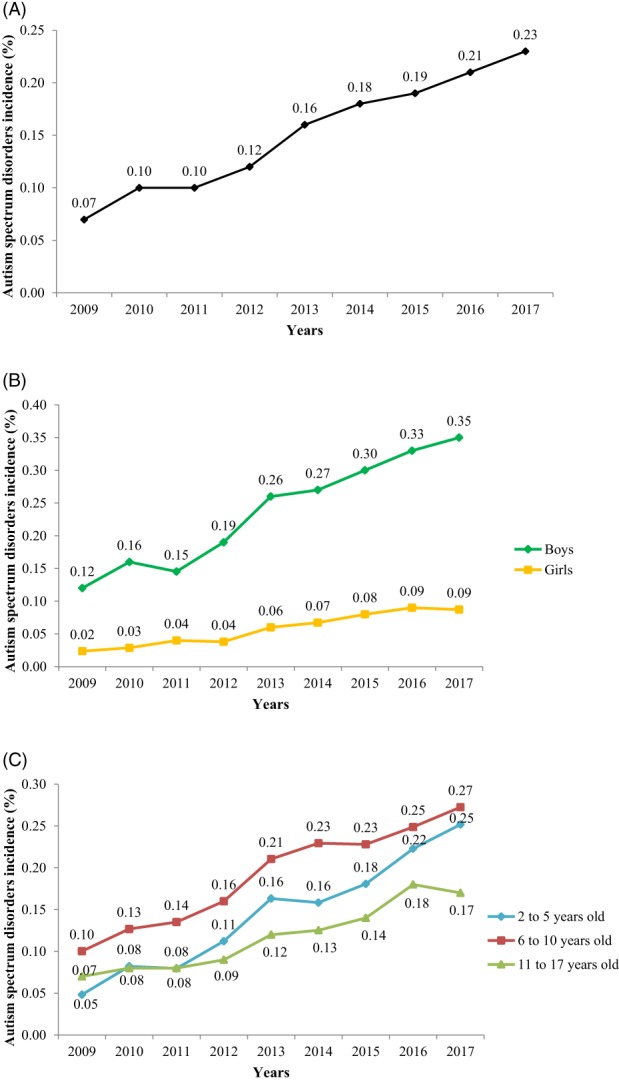
Autism spectrum disorders diagnosis incidence rate (%) among children from 2 to 17 years old between 2009 and 2017 in Catalonia region, Spain. (**A**) Overall autism spectrum disorders diagnosis incidence rate (%). (**B**) Autism spectrum disorders diagnosis incidence rate (%) stratified by sex. (**C**) Autism spectrum disorders diagnosis incidence rate (%) stratified by age group at diagnosis.

**Figure 3:**
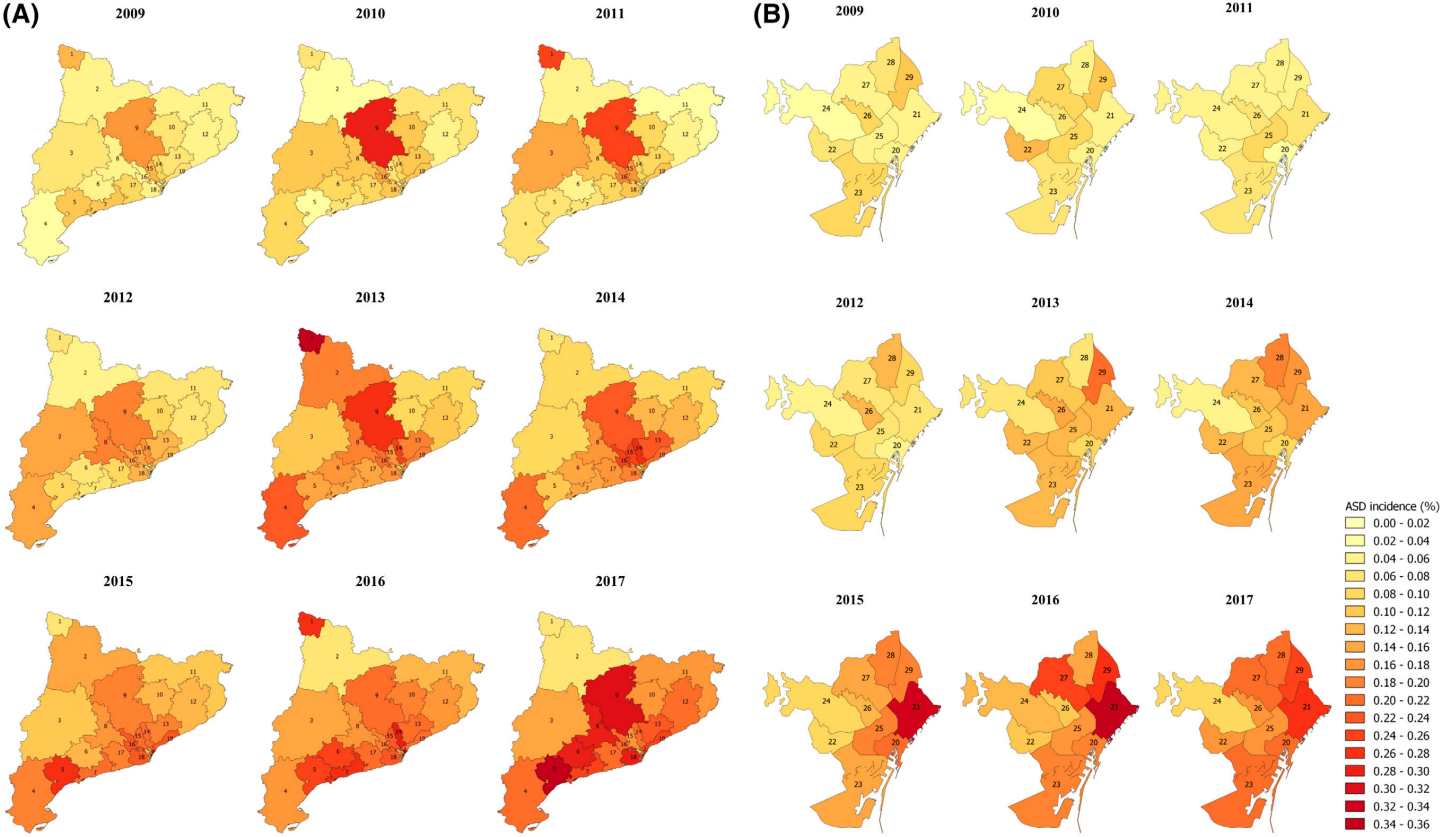
Autism spectrum disorders diagnosis incidence rate (%) among children from 2 to 17 years old between 2009 and 2017 stratified by healthcare areas of Catalonia region, Spain. **(A)** Healthcare areas of Catalonia (excluding Barcelona city). **(B)** Healthcare areas of Barcelona city. **1**: Aran; **2**: Alt Pirineu; **3**: Lleida; **4**: Terres de l'Ebre; **5**: Baix Camp–Priorat; **6**: Alt Camp–Conca de Barberà; **7**: Tarragonés–Baix Penedès; **8**: Anoia; **9**: Solsonès–Bages–Berguedà; **10**: Osona; **11**: Girona Nord; **12**: Girona Sud; **13**: Vallès Oriental; **14**: Vallès Occidental Est; **15**: Vallès Occidental Oest; **16**: Baix Llobregat Nord; **17**: Alt Penedès–Garraf; **18**: Baix Llobregat Centre‐Litoral i L'Hospitalet de Llobregat; **19**: Barcelonès Nord i Maresme; **20**: Barcelona Ciutat Vella; **21**: Barcelona Sant Martí; **22**: Barcelona les Corts; **23**: Barcelona Sants–Montjuïc; **24**: Barcelona Sarrià–Sant Gervasi; **25**: Barcelona Eixample; **26**: Barcelona Gràcia; **27**: Barcelona Horta–Guinardó; **28**: Barcelona Nou Barris; **29**: Barcelona Sant Andreu. Abbreviation: ASD, autism spectrum disorders.

During the period between 2009 and 2017, there was a significant increase in the overall ASD diagnosis incidence from 0.07% (95% CI 0.06–0.09) in 2009 to 0.23% (95% CI 0.21–0.24) in 2017 (Table 1 and Fig. 2A). ASD diagnosis incidence increased linearly by 15% (95% CI 1.14–1.15) each year. Remarkably, we observed a greater increase in diagnosis incidence between 2012 (0.12%) and 2013 (0.16%) than in other years of the study period. When stratified by sex, we observed a greater increase in ASD diagnosis incidence in girls (0.02% in 2009 and 0.09% in 2017) than in boys. However, throughout the entire study period, the ASD diagnosis incidence remained higher in boys than in girls. In addition, there was a significant increase in diagnosis incidence between 2012 and 2013 in boys that was not detected in girls (Table 1 and Fig. 2B). Regarding the age groups at diagnosis, we observed a greater increase in ASD diagnosis incidence in 2‐ to 5‐year‐olds (0.05% in 2009 and 0.25% in 2017) than in the other age groups (Table 1 and Fig. 2C). However, throughout the entire period between 2009 and 2017, more children were diagnosed with ASD in the 6–10 years age group than in the two other age groups at the time of diagnosis. The age group with the fewest children diagnosed with ASD was the 11–17 years old group. The mean age of ASD diagnosis was estimated to be 8.8 years, and the median was 8 years. When studying ASD diagnosis incidence by healthcare area, we noted an increase between 2009 and 2017 in all areas, although there was no clear pattern across healthcare areas (Fig. 3 and Supporting Information Table S1). During the study period, when taking the ASD incidence within each healthcare area separately, the incidence varied significantly in most of the healthcare areas of Catalonia, except in Alt Camp–Conca de Barberà, Osona, Aran, Barcelona Ciutat Vella, Barcelona les Corts, Barcelona Sants, Barcelona Eixample, Barcelona Gràcia, Barcelona Horta, and Barcelona NouBarris. However, when assessing differences in the annual diagnosis incidence between healthcare areas, no difference across them was found. Similarly, there were no significant differences by sex and age group between healthcare areas from 2009 to 2017 (Supporting Information Fig. S1).

## Discussion

The present study has assessed the ASD prevalence in 2017 and the ASD diagnosis incidence between 2009 and 2017, as well as their temporal and geographical variability, among children aged 2–17 years in Catalonia region in Spain. The ASD prevalence in 2017 was estimated at 1.23%, with a sex ratio of 4.5 (boy:girl), and with the highest prevalence observed in 11‐ to 17‐year‐olds. The incidence of new ASD diagnosed cases increased significantly between 2009 and 2017, from 0.07% to 0.23%, with a greater increase in girls and, notably, in those aged 2–5 years at the time of diagnosis. We also observed differences in the ASD prevalence between geographical regions in 2017, but not in the annual diagnosis incidence across the period studied. When stratified by sex and age group, there were no significant differences between geographical regions in the annual diagnosis incidence over the study period.

Prior to 1990, the first studies on ASD prevalence in children reported a prevalence of 0.05% [Lotter, 1966], which has since increased dramatically. Studies carried out between 2000 and 2016 showed a median worldwide ASD prevalence of 0.70% [Baxter et al., 2015; Elsabbagh et al., 2012] and a median ASD prevalence of 0.61% in Europe [Elsabbagh et al., 2012; Fortea Sevilla et al., 2013; Idring et al., 2015; Saemundsen et al., 2013; Skonieczna‐Żydecka et al., 2017; van Bakel et al., 2015]. Among the most recent European studies, the highest ASD prevalence was found in Spain with an estimate of 1.55% among preschool children [Morales‐Hidalgo et al., 2018]. In contrast, the lowest ASD prevalence was found in Poland with an overall estimate of 0.35% among children up to 18 years old in 2014 [Skonieczna‐Żydecka et al., 2017]. The methodologies applied in the different studies differ in case definition, identification, and diagnosis, making it difficult to compare studies. Most of the studies of ASD prevalence include population‐based screening studies, which are based on samples that are not always representative of the reference population and would therefore provide biased estimations. Other studies, particularly those including large populations, have used different strategies or datasets to define ASD cases, including parental reports, healthcare provider datasets, special educational datasets, or national registers. Therefore, when interpreting and comparing studies on ASD prevalence it is crucial to keep in mind the limitations inherent to each study design in order to draw meaningful conclusions.

In our study, the observed sex ratio of 4.5 (boy:girl) is in agreement with the median ratio of 4.0 reported in most epidemiological studies of ASD prevalence [American Psychiatric Association, 2013]. Notwithstanding that, this ratio varies considerably between studies, ranging from 1.0 [Chaaya et al., 2016] to 15.7 [Baird et al., 2006]. The causes of the observed male bias in ASD prevalence are not completely understood, but may include genetic factors or differences in detection and diagnosis. Some studies have demonstrated that girls could be protected from inherited and de novo genetic risk variants for ASD [Constantino, Zhang, Frazier, Abbacchi, & Law, 2010; Sebat et al., 2007]. Others suggest that genes on the sex chromosomes and sex hormones, particularly testosterone, may modulate the effects of genetic variation on the appearance of an autistic phenotype [Auyeung et al., 2009; Baron‐Cohen et al., 2011; Werling & Geschwind, 2013]. Differences in detection and diagnosis of ASD might be due to the fact that girls with ASD show higher social motivation and a greater capacity for traditional friendships than boys with ASD [Bargiela, Steward, & Mandy, 2016; Sedgewick, Hill, Yates, Pickering, & Pellicano, 2016]. Moreover, girls also score lower on measures of repetitive and stereotyped behavior, which might lead to an underdiagnosis of ASD [Rubenstein, Wiggins, & Lee, 2015].

Early diagnosis of ASD is critical, but it is often delayed until school age. Evidence suggests that interventions to improve the functioning in ASD children may be more effective in younger patients, and early treatment improves long‐term prognosis [Mandell, 2005]. ASD symptoms are typically recognized during the second year of life (12–24 months), but the disorder can be diagnosed either before 12 months, if developmental symptoms are severe, or after 24 months, if symptoms are more subtle [American Psychiatric Association, 2013]. In our study, the highest ASD prevalence was observed in 11‐ to 17‐year‐olds, with a mean age at diagnosis of 8.8 years, and a median of 8 years. Our findings are consistent with another study conducted in children up to 17 years old in Sweden, which found that the ASD prevalence increased with age, with a higher ASD prevalence among teenagers and a median age at diagnosis of 8 years [Idring et al., 2015]. However, the results of our study differ from those of other studies that compared ASD prevalence between age groups [Al‐Farsi et al., 2011; Davidovitch et al., 2013; Latif & Williams, 2007; May et al., 2017; Sheldrick, Maye, & Carter, 2017; Skonieczna‐Żydecka et al., 2017; Yeargin‐Allsopp et al., 2003]. Globally, these studies found that ASD prevalence was higher among 5‐ to 9‐year‐olds, and that the mean age of diagnosis was ~5.5 years. In two additional studies, the ASD prevalence did not differ between age groups [Harrison, 2005; Kogan et al., 2009]. The lower prevalence rates in younger children in our study could be due to clinicians' reluctance to label children as having ASD at very young ages, or a lack of training among health professionals in diagnosing ASD at early ages. Nevertheless, our temporal trend data suggest that ASD diagnosis at early ages has improved over the study period.

We found a significant threefold increase in overall ASD diagnosis incidence between 2009 and 2017, consistent with trends in other countries [Davidovitch et al., 2013; Jensen et al., 2014; Nassar et al., 2009; Pelly et al., 2015; Raz et al., 2015]. This increase could be due to etiologic factors, but is most likely related to non‐etiologic factors; changes in the definition of ASD during the period could explain the observed overall increase in the incidence of ASD diagnosis. In the last edition of the Diagnostic and Statistical Manual of mental disorders (DSM) [American Psychiatric Association, 2013], ASD included several disorders that were not included in the previous editions, which would explain that more children could be diagnosed as having ASD. Another possible explanation could be the increasing awareness of ASD in recent years among parents, teachers, psychiatrists, and pediatricians. In the region studied, several professional training actions in diagnostic tools for ASD (Autism Diagnostic Interview Revised (ADI‐R) and Autism Diagnostic Observational Schedule [ADOS]) have been addressed to professionals working in public mental healthcare centers since 2011. In addition, the Catalan Health Department implemented a comprehensive plan in some geographical areas to improve ASD detection, diagnosis, and treatment. However, the data showed that ASD diagnosis incidence still increases each year, which means that the diagnostic stability of ASD has not yet been achieved. Therefore, there is a need to invest more resources to improve ASD diagnosis, especially at early ages.

The main strengths of our study are the design, which is based on administrative data, and the large sample size included. It is one of the few studies carried out in Southern Europe based on register data including a large annual number of births (80,000 per year approximately) [Idescat, 2018]. The last studies conducted in Spain based on multistage screening approach have the advantage of detecting and evaluating the cases of ASD with better precision but they have the limitation that only a point prevalence rate can be estimated. In our study, the use of administrative data has allowed us to estimate the prevalence, temporal trends of the diagnosis incidence as well as the geographical variability for both prevalence and incidence. Our study also included data from a relatively long time period, from 2009 to 2017, allowing a thorough analysis of temporal trends. Finally, we studied the differences in prevalence and incidence of ASD diagnosis for each sex, age group, and across geographical healthcare areas. However, we acknowledge that the small numbers in the incidence of ASD diagnosis across geographical healthcare areas might have limited our capability of detecting differences.

Our study has also some limitations. The main limitation of our study is its retrospective nature: studies based on registers only capture individuals who accessed the healthcare service, rather than sampling from the population at large. As a result, participants with the studied disorder who are not in contact with these services are yet unidentified and not included as cases, which may lead to underestimation of ASD diagnosis prevalence and incidence rates. While we recognize these limitations, other study designs such as the methods of case confirmation have also important limitations including a low participation of families in the study that would lead to bias estimations. On the contrary, the use of data registers is less costly and more time‐efficient and may provide a more complete coverage of a large population. Furthermore, Spain's National Health System provides universal access to health services for all native and foreign‐born children [García‐Altés et al., 2018; García‐Armesto et al., 2010]. Some people also have health cover from private insurers, which covers services offered by the public system, as well as services beyond those offered by the public system (e.g., elective surgery or dental care). This provides easier individual direct access to healthcare, more personalized care, shorter waiting lists, and private rooms in private hospitals [Fusté, Séculi, Brugulat, Medina, & Juncà, 2005]. Therefore, the double coverage allows the use of public services and, at the same time, those that are offered by private insurers according to the user's preferences and needs; thus, this private cover supplements the public system. In Catalonia, the rate of this double cover is much higher than in other Spanish regions, estimated to reach about 25% of the population [Direcció General de Planificació en Salut, 2016]. The remaining 75% of the population is covered only by the public health system. Therefore, we expect to have a low number of ASD diagnosed cases not included in our study, which would lead to a slight underestimation of prevalence and incidence estimates found in our study. In addition, study design masked variation in ASD diagnostic practices. As our study is based on register data, we cannot confirm that ASD diagnosis assessment has been made in the standard way for all ASD cases. While some clinicians work within a multidisciplinary team, others work independently to make a diagnostic decision. This fact could partly explain the variability in ASD diagnosis prevalence and incidence between healthcare areas. Nevertheless, in all the public mental healthcare centers of Catalonia where the diagnosis of ASD could be made, there is at least one professional specialized in ADI‐R and ADOS tools as clinical guidelines recommended. This fact, however, does not ensure that the ASD diagnosis would be done using these diagnostic tools. As an example, the time constraints for each visit in the public mental healthcare centers can lead to the health professionals to perform shorter diagnosis based on the DSM criteria instead of ADI‐R and ADOS. Additionally, no efforts are made by the data provider to perform a case validation of the ASD cases included in the register. For future studies on the epidemiological monitoring of ASD based on this data, a case validation study should be done as well as an evaluation of the completeness of the data. It is also possible that ASD diagnosis was underestimated if children were first diagnosed with an attention deficit and hyperactivity disorder, which occurs concomitantly in 30% to 50% of children with ASD [Davis & Kollins, 2012]. Finally, we only retrieved information on ASD cases from the public healthcare centers that are under the Catalan Health Department. We were unable to access data from private healthcare centers, the Child Development and Early Care Centers that are under the Catalan Welfare Department, and the Psycho‐Pedagogical Assessment Teams that are under the Catalan Educational Department. However, in Catalonia there is a health program called “Healthy Childhood” aimed at children aged 0–14 years old [Direcció General de Salut Pública, 2008]. This program is about issues related to childhood health (physical and psychomotor development, routine immunizations, locomotor system, genitourinary system, ophthalmology, otorhinolaryngology, oral health, etc.) and visits to the pediatrician are scheduled throughout the whole childhood. Although it is estimated that a maximum of 25% of the population has double health coverage [Direcció General de Planificació en Salut, 2016], it is important to point out that most of the children insured in the Catalan Health System follow this program. Therefore, we expect that most of these ASD diagnosed cases would also had at least one contact with the public healthcare centers in relation to their ASD diagnosis or for other reasons and notify the pediatrician about the diagnosis, that is, at a regular visits within the “Healthy childhood” program, and that their ASD diagnosis would be registered. Although the age of diagnosis might not be accurately registered for these cases, we were able to include them in the overall ASD estimations. Future studies should focus on harmonizing and merging the data from all existing registers to provide a more accurate estimate of ASD diagnosis prevalence and incidence.

In conclusion, in Catalonia region, we found an ASD prevalence of 1.23% in 2017. This prevalence was higher in boys than in girls (sex ratio 4.5), and was highest in the 11–17 years age group. We also observed a threefold increase in ASD diagnosis incidence between 2009 and 2017, and that this was more pronounced among girls and in children aged 2–5 years at the time of diagnosis. We observed geographical differences in the ASD prevalence in 2017 between healthcare areas, but not for the annual ASD diagnosis incidence during the period 2009 and 2017. This study provides new epidemiological information on the prevalence and incidence of ASD diagnosis in children in Catalonia region, and is one of the few studies that has been carried out in the South of Europe. The data collected here will add to the available information for planning and evaluating the needs of the healthcare services in Catalonia, especially those related to early diagnosis and intervention. Finally, it is also important to point out to the need to establish improved methods of registering the diagnosis of ASD to ensure that the health burden of the condition is accurately measured.

## Conflict of Interest

A.T. is the father of a child with ASD and a member of two patient associations (Aprenem and JuntsAutisme) that strive for the inclusion of people with ASD.

## Supporting information


**Table S1.** Autism spectrum disorders prevalence rate (%) in 2017 and ASD diagnosis incidence rates (%) between 2009 and 2017 among children between 2 to 17 years old by healthcare areas of the Catalonia region, Spain.
**Figure S1**. Incidence risk ratio of autism spectrum disorders diagnosis incidence over the study period from 2009 to 2017 between healthcare areas of the Catalonia region, Spain, stratified by sex and age group at diagnosis. **(A)** Boys from 2 to 5 years old **(B)** Boys from 6 to 10 years old **(C)** Boys from 11 to 17 years old **(D)** Girls from 2 to 5 years old **(E)** Girls from 6 to 10 years old **(F)** Girls from 11 to 17 years old.
