# Supplementary material for: Temporal and Geographical Variability of Prevalence and Incidence of Autism Spectrum Disorder Diagnoses in Children in Catalonia, Spain
Source: Autism Res. 2019 Jul 17;12(11):1693–705. doi: 10.1002/aur.2172 (PMC6900126; doi:10.1002/aur.2172)
Supplement: Supplementary file 1 — Table S1. Autism spectrum disorders prevalence rate (%) in 2017 and ASD diagnosis incidence rates (%) between 2009 and 2017 among children between 2 to 17 years old by healthcare areas of the Catalonia region, Spain. Figure S1. Incidence risk ratio of autism spectrum disorders diagnosis incidence over the study period from 2009 to 2017 between healthcare areas of the Catalonia region, Spain, stratified by sex and age group at diagnosis. (A) Boys from 2 to 5 years old (B) Boys from 6 to 10 years old (C) Boys from 11 to 17 years old (D) Girls from 2 to 5 years old (E) Girls from 6 to 10 years old (F) Girls from 11 to 17 years old. [file AUR-12-1693-s001.docx]

**Supplementary Material**

**Temporal and geographical variability of prevalence and incidence of autism spectrum disorder diagnoses in children in Catalonia, Spain**

Laura Pérez-Crespo, Albert Prats-Uribe, Aurelio Tobias, Enric Duran-Tauleria, Ricard Coronado, Amaia Hervás, Mònica Guxens

**Table S1**. Autism spectrum disorders prevalence rate (%) in 2017 and ASD diagnosis incidence rates (%) between 2009 and 2017 among children between 2 to 17 years old by healthcare areas of the Catalonia region, Spain…………………………………………………………………………….. 2

**Figure S1**. Incidence risk ratio of autism spectrum disorders diagnosis incidence over the study period from 2009 to 2017 between healthcare areas of the Catalonia region, Spain, stratified by sex and age group at diagnosis. **(A)** Boys from 2 to 5 years old **(B)** Boys from 6 to 10 years old **(C)** Boys from 11 to 17 years old **(D)** Girls from 2 to 5 years old **(E)** Girls from 6 to 10 years old **(F)** Girls from 11 to 17 years old………………………………………………………………………………..7

^†^ Aran healthcare area (Healthcare area number 1) was excluded due to the low population denominator and number of autism spectrum disorder cases

**2**: Alt Pirineu; **3**: Lleida; **4**: Terres de l’Ebre; **5**: Baix Camp- Priorat; **6**: Alt Camp- Conca de Barberà; **7**: Tarragonés- Baix Penedès; **8**: Anoia; **9**: Solsonès- Bages- Berguedà; **10**: Osona; **11**: Girona Nord; **12**: Girona Sud; **13**: Vallès Oriental; **14**: Vallès Occidental Est; **15**: Vallès Occidental Oest; **16**: Baix Llobregat Nord; **17**: Alt Penedès - Garraf; **18**: Baix Llobregat Centre-Litoral i L’Hospitalet de Llobregat; **19**: Barcelonès Nord i Maresme; **20**: Barcelona Ciutat Vella; **21**: Barcelona Sant Martí; **22**: Barcelona les Corts; **23**: Barcelona Sants- Montjuïc; **24**: Barcelona Sarrià- Sant Gervasi; **25**: Barcelona Eixample; **26**: Barcelona Gràcia; **27**: Barcelona Horta- Guinardó; **28**: Barcelona Nou Barris; **29**: Barcelona Sant Andreu.

ASD=autism spectrum disorders

**Table S1. Autism spectrum disorders prevalence rate (%) in 2017 and ASD diagnosis incidence rate (%) between 2009 and 2017 among children between 2 to 17 years old by healthcare areas of the Catalonia region**

|  | **Prevalence** |  | **Incidence** | | | | | | | |  |
| --- | --- | --- | --- | --- | --- | --- | --- | --- | --- | --- | --- |
| **Healthcare area** | **2017** |  | **2009** | **2010** | **2011** | **2012** | **2013** | **2014** | **2015** | **2016** | **2017** |
| **Lleida** |  |  |  |  |  |  |  |  |  |  |  |
| N | 596 |  | 45 | 66 | 84 | 91 | 68 | 56 | 68 | 92 | 89 |
| Rate  (95% CI) | 1.00  (0.99; 1.00) |  | 0.08  (0.00; 0.16) | 0.11  (0.04; 0.19) | 0.14  (0.07; 0.22) | 0.15  (0.08; 0.22) | 0.11  (0.04; 0.19) | 0.09  (0.02; 0.17) | 0.11  (0.04; 0.19) | 0.15  (0.08; 0.23) | 0.15  (0.07; 0.22) |
| **Alt Camp-Conca de Barberà** |  |  |  |  |  |  |  |  |  |  |  |
| N | 118 |  | 5 | 9 | 6 | 7 | 18 | 16 | 14 | 27 | 31 |
| Rate  (95% CI) | 1.14  (0.95; 1.36) |  | 0.05  (-0.14; 0.24) | 0.09  (-0.10; 0.27) | 0.06  (-0.13; 0.24) | 0.07  (-0.12; 0.25) | 0.17  (0.00; 0.35) | 0.15  (-0.02; 0.33) | 0.13  (-0.04; 0.31) | 0.26  (0.09; 0.42) | 0.30  (0.14; 0.46) |
| **Tarragonès - Baix Penedès** |  |  |  |  |  |  |  |  |  |  |  |
| N | 772 |  | 63 | 41 | 47 | 41 | 93 | 94 | 129 | 164 | 158 |
| Rate  (95% CI) | 1.27  (1.18; 1.36) |  | 0.11  (0.03; 0.19) | 0.07  (-0.01; 0.15) | 0.08  (0.00; 0.15) | 0.07  (-0.01; 0.14) | 0.15  (0.08; 0.23) | 0.15  (0.08; 0.23) | 0.21  (0.14; 0.28) | 0.27  (0.20; 0.34) | 0.26  (0.19; 0.33) |
| **Baix Camp-Priorat** |  |  |  |  |  |  |  |  |  |  |  |
| N | 466 |  | 38 | 12 | 25 | 37 | 52 | 42 | 98 | 90 | 124 |
| Rate  (95% CI) | 1.30  (1.19; 1.42) |  | 0.11  (0.01; 0.20) | 0.03  (-0.07; 0.13) | 0.07  (-0.03; 0.17) | 0.10  (0.00; 0.20) | 0.14  (0.05; 0.23) | 0.11  (0.02; 0.21) | 0.27  (0.18; 0.36) | 0.25  (0.16; 0.34) | 0.35  (0.26; 0.43) |
| **Terres de l' Ebre** |  |  |  |  |  |  |  |  |  |  |  |
| N | 341 |  | 8 | 28 | 20 | 47 | 68 | 63 | 54 | 51 | 57 |
| Rate  (95% CI) | 1.21  (1.09; 1.35) |  | 0.03  (-0.09; 0.14) | 0.09  (-0.01; 0.20) | 0.07  (-0.04; 0.18) | 0.16  (0.05; 0.26) | 0.23  (0.13; 0.33) | 0.21  (0.11; 0.31) | 0.19  (0.08; 0.29) | 0.18  (0.07; 0.28) | 0.20  (0.10; 0.31) |
| **Girona Nord** |  |  |  |  |  |  |  |  |  |  |  |
| N | 434 |  | 29 | 37 | 22 | 43 | 48 | 53 | 67 | 82 | 95 |
| Rate  (95% CI) | 0.74  (0.70; 0.78) |  | 0.05  (-0.03; 0.13) | 0.06  (-0.01; 0.14) | 0.04  (-0.04; 0.12) | 0.07  (0.00; 0.15) | 0.08  (0.00; 0.16) | 0.09  (0.01; 0.17) | 0.11  (0.04; 0.19) | 0.14  (0.06; 0.21) | 0.16  (0.09; 0.24) |
| **Girona Sud** |  |  |  |  |  |  |  |  |  |  |  |
| N | 814 |  | 40 | 45 | 47 | 58 | 96 | 120 | 125 | 156 | 182 |
| Rate  (95% CI) | 0.90  (0.88; 0.92) |  | 0.05  (-0.02; 0.11) | 0.05  (-0.01; 0.11) | 0.05  (-0.01; 0.12) | 0.06  (0.00; 0.13) | 0.11  (0.04; 0.17) | 0.13  (0.07; 0.19) | 0.14  (0.08; 0.20) | 0.17  (0.11; 0.23) | 0.20  (0.14; 0.26) |

**Table S1 (continuation). Autism spectrum disorders prevalence rate (%) in 2017 and ASD diagnosis incidence rate (%) between 2009 and 2017 among children between 2 to 17 years old by healthcare areas of the Catalonia region**

|  | **Prevalence** |  | **Incidence** | | | | | | | |  |
| --- | --- | --- | --- | --- | --- | --- | --- | --- | --- | --- | --- |
| **Healthcare area** | **2017** |  | **2009** | **2010** | **2011** | **2012** | **2013** | **2014** | **2015** | **2016** | **2017** |
| **Anoia** |  |  |  |  |  |  |  |  |  |  |  |
| N | 275 |  | 10 | 21 | 22 | 40 | 39 | 36 | 34 | 35 | 59 |
| Rate  (95% CI) | 1.35  (1.20; 1.51) |  | 0.05  (-0.09; 0.19) | 0.10  (-0.03; 0.23) | 0.11  (-0.02; 0.24) | 0.19  (0.07; 0.32) | 0.19  (0.07; 0.31) | 0.17  (0.05; 0.30) | 0.17  (0.04; 0.29) | 0.17  (0.05; 0.30) | 0.29  (0.17; 0.40) |
| **Osona** |  |  |  |  |  |  |  |  |  |  |  |
| N | 261 |  | 19 | 30 | 23 | 26 | 27 | 38 | 34 | 55 | 48 |
| Rate  (95% CI) | 0.93  (0.90; 0.96) |  | 0.07  (-0.04; 0.18) | 0.11  (0.00; 0.22) | 0.08  (-0.03; 0.19) | 0.09  (-0.02; 0.20) | 0.10  (-0.02; 0.21) | 0.13  (0.03; 0.24) | 0.12  (0.01; 0.23) | 0.19  (0.09; 0.30) | 0.17  (0.06; 0.28) |
| **Solsonès- Bages- Berguedà** |  |  |  |  |  |  |  |  |  |  |  |
| N | 755 |  | 64 | 111 | 98 | 81 | 115 | 92 | 79 | 87 | 126 |
| Rate  (95% CI) | 1.84  (1.71; 1.98) |  | 0.16  (0.07; 0.25) | 0.28  (0.20; 0.36) | 0.24  (0.16; 0.33) | 0.20  (0.11; 0.28) | 0.28  (0.20; 0.36) | 0.22  (0.14; 0.31) | 0.19  (0.10; 0.28) | 0.21  (0.13; 0.30) | 0.31  (0.23; 0.39) |
| **Aran** |  |  |  |  |  |  |  |  |  |  |  |
| N | 19 |  | 2 | 1 | 4 | 1 | 5 | 1 | 1 | 4 | 1 |
| Rate  (95% CI) | 1.32  (0.79; 1.93) |  | 0.12  (-0.33; 0.58) | 0.06  (-0.41; 0.53) | 0.25  (-0.17;0.68) | 0.07  (-0.42; 0.55) | 0.35  (-0.07; 0.77) | 0.07  (-0.43; 0.56) | 0.07  (-0.43; 0.57) | 0.27  (-0.16; 0.71) | 0.07  (-0.43; 0.57) |
| **Alt Pirineu** |  |  |  |  |  |  |  |  |  |  |  |
| N | 64 |  | 4 | 3 | 4 | 5 | 18 | 9 | 13 | 7 | 7 |
| Rate  (95% CI) | 0.71  (0.60; 0.82) |  | 0.04  (-0.15; 0.24) | 0.03  (-0.17; 0.23) | 0.04  (-0.15; 0.23) | 0.05  (-0.14; 0.25) | 0.19  (0.01; 0.37) | 0.10  (-0.10; 0.29) | 0.14  (-0.05; 0.33) | 0.08  (-0.12; 0.27) | 0.08  (-0.12; 0.28) |
| **Baix Llobregat Nord** |  |  |  |  |  |  |  |  |  |  |  |
| N | 442 |  | 13 | 47 | 56 | 44 | 64 | 80 | 72 | 59 | 65 |
| Rate  (95% CI) | 1.40  (1.28; 1.54) |  | 0.04  (-0.07; 0.16) | 0.16  (0.05; 0.26) | 0.18  (0.08; 0.28) | 0.14  (0.04; 0.24) | 0.20  (0.11; 0.30) | 0.25  (0.16; 0.35) | 0.23  (0.13; 0.33) | 0.19  (0.09; 0.29) | 0.21  (0.11; 0.30) |
| **Vallès Oriental** |  |  |  |  |  |  |  |  |  |  |  |
| N | 1090 |  | 62 | 85 | 74 | 112 | 159 | 194 | 157 | 178 | 188 |
| Rate  (95% CI) | 1.32  (1.24; 1.40) |  | 0.08  (0.01; 0.14) | 0.10  (0.04; 0.17) | 0.09  (0.02; 0.15) | 0.14  (0.07; 0.20) | 0.19  (0.13; 0.25) | 0.23  (0.17; 0.29) | 0.19  (0.13; 0.25) | 0.21  (0.15; 0.28) | 0.23  (0.17; 0.29) |

**Table S1 (continuation). Autism spectrum disorders prevalence rate (%) in 2017 and ASD diagnosis incidence rate (%) between 2009 and 2017 among children between 2 to 17 years old by healthcare areas of the Catalonia region**

|  | **Prevalence** |  | **Incidence** | | | | | | | |  |
| --- | --- | --- | --- | --- | --- | --- | --- | --- | --- | --- | --- |
| **Healthcare area** | **2017** |  | **2009** | **2010** | **2011** | **2012** | **2013** | **2014** | **2015** | **2016** | **2017** |
| **Vallès Occidental oest** |  |  |  |  |  |  |  |  |  |  |  |
| N | 1166 |  | 106 | 64 | 135 | 116 | 134 | 204 | 169 | 190 | 150 |
| Rate  (95% CI) | 1.40  (1.32; 1.48) |  | 0.13  (0.07; 0.20) | 0.08  (0.01; 0.14) | 0.16  (0.10; 0.23) | 0.14  (0.08; 0.20) | 0.16  (0.10; 0.22) | 0.24  (0.19; 0.30) | 0.20  (0.14; 0.26) | 0.23  (0.17; 0.29) | 0.18  (0.12; 0.24) |
| **Vallès Occidental est** |  |  |  |  |  |  |  |  |  |  |  |
| N | 1141 |  | 67 | 71 | 60 | 130 | 181 | 200 | 165 | 215 | 166 |
| Rate (95% CI) | 1.51  (1.42; 1.60) |  | 0.09  (0.02; 0.16) | 0.10  (0.03; 0.17) | 0.08  (0.01; 0.15) | 0.17  (0.11; 0.24) | 0.24  (0.18; 0.30) | 0.26  (0.20; 0.33) | 0.22  (0.16; 0.28) | 0.28  (0.22; 0.34) | 0.22  (0.16; 0.28) |
| **Barcelona Ciutat Vella** |  |  |  |  |  |  |  |  |  |  |  |
| N | 103 |  | 7 | 7 | 5 | 7 | 13 | 12 | 25 | 24 | 24 |
| Rate  (95% CI) | 0.88  (0.81; 0.94) |  | 0.05  (-0.11; 0.21) | 0.05  (-0.11; 0.21) | 0.03  (-0.13; 0.19) | 0.05  (-0.11; 0.21) | 0.10  (-0.06; 0.26) | 0.09  (-0.07; 0.25) | 0.20  (0.05; 0.36) | 0.20  (0.04; 0.36) | 0.20  (0.04; 0.37) |
| **Barcelona Sant Martí** |  |  |  |  |  |  |  |  |  |  |  |
| N | 446 |  | 15 | 20 | 27 | 25 | 42 | 52 | 111 | 118 | 90 |
| Rate  (95% CI) | 1.32  (1.20; 1.44) |  | 0.04  (-0.06; 0.15) | 0.06  (-0.04; 0.16) | 0.08  (-0.02; 0.18) | 0.07  (-0.03; 0.18) | 0.12  (0.02; 0.22) | 0.15  (0.06; 0.25) | 0.33  (0.24; 0.42) | 0.35  (0.26; 0.43) | 0.27  (0.17; 0.36) |
| **Barcelona les Corts** |  |  |  |  |  |  |  |  |  |  |  |
| N | 84 |  | 8 | 13 | 6 | 9 | 13 | 13 | 9 | 12 | 17 |
| Rate  (95% CI) | 0.83  (0.75; 0.91) |  | 0.07  (-0.11; 0.25) | 0.12  (-0.06; 0.30) | 0.06  (-0.13; 0.24) | 0.09  (-0.10; 0.27) | 0.13  (-0.05; 0.31) | 0.13  (-0.05; 0.31) | 0.09  (-0.10; 0.27) | 0.12  (-0.06; 0.30) | 0.17  (-0.01; 0.35) |
| **Barcelona Sants - Montjuïc** |  |  |  |  |  |  |  |  |  |  |  |
| N | 254 |  | 21 | 18 | 16 | 24 | 29 | 37 | 36 | 45 | 49 |
| Rate  (95% CI) | 1.11  (0.98; 1.26) |  | 0.08  (-0.03; 0.20) | 0.07  (-0.05; 0.19) | 0.06  (-0.06; 0.18) | 0.10  (-0.02; 0.22) | 0.12  (0.00; 0.24) | 0.16  (0.04; 0.28) | 0.16  (0.04; 0.28) | 0.20  (0.08; 0.31) | 0.21  (0.10; 0.33) |
| **Barcelona Sarrià - Sant Gervasi** |  |  |  |  |  |  |  |  |  |  |  |
| N | 131 |  | 5 | 7 | 14 | 11 | 17 | 14 | 23 | 33 | 23 |
| Rate  (95% CI) | 0.55  (0.46; 0.64) |  | 0.02  (-0.10; 0.14) | 0.03  (-0.09; 0.15) | 0.06  (-0.06; 0.18) | 0.05  (-0.08; 0.17) | 0.07  (-0.05; 0.19) | 0.06  (-0.06; 0.18) | 0.10  (-0.02; 0.22) | 0.14  (0.02; 0.26) | 0.10  (-0.02; 0.22) |

**Table S1 (continuation). Autism spectrum disorders prevalence rate (%) in 2017 and ASD diagnosis incidence rate (%) between 2009 and 2017 among children between 2 to 17 years old by healthcare areas of the Catalonia region**

|  | **Prevalence** |  | **Incidence** | | | | | | | |  |
| --- | --- | --- | --- | --- | --- | --- | --- | --- | --- | --- | --- |
| **Healthcare area** | **2017** |  | **2009** | **2010** | **2011** | **2012** | **2013** | **2014** | **2015** | **2016** | **2017** |
| **Barcelona Eixample** |  |  |  |  |  |  |  |  |  |  |  |
| N | 300 |  | 18 | 32 | 33 | 25 | 45 | 37 | 61 | 53 | 54 |
| Rate  (95% CI) | 0.96  (0.94; 0.98) |  | 0.05  (-0.05; 0.15) | 0.09  (-0.01; 0.19) | 0.10  (0.00; 0.20) | 0.08  (-0.03; 0.18) | 0.14  (0.04; 0.24) | 0.12  (0.01; 0.22) | 0.19  (0.09; 0.29) | 0.17  (0.07; 0.27) | 0.17  (0.07; 0.27) |
| **Barcelona Gràcia** |  |  |  |  |  |  |  |  |  |  |  |
| N | 133 |  | 14 | 11 | 10 | 21 | 23 | 16 | 19 | 17 | 26 |
| Rate  (95% CI) | 0.87  (0.81; 0.93) |  | 0.09  (-0.06; 0.24) | 0.07  (-0.08; 0.22) | 0.06  (-0.09; 0.21) | 0.14  (-0.01; 0.28) | 0.15  (0.01; 0.30) | 0.11  (-0.05; 0.26) | 0.13  (-0.02; 0.28) | 0.11  (-0.04; 0.26) | 0.17  (0.03; 0.31) |
| **Barcelona Horta - Guinardó** |  |  |  |  |  |  |  |  |  |  |  |
| N | 242 |  | 13 | 23 | 11 | 15 | 23 | 28 | 35 | 58 | 45 |
| Rate  (95% CI) | 1.08  (0.96; 1.23) |  | 0.05  (-0.07; 0.18) | 0.10  (-0.02; 0.22) | 0.05  (-0.08; 0.17) | 0.06  (-0.06; 0.19) | 0.10  (-0.02; 0.22) | 0.12  (0.00; 0.25) | 0.16  (0.04; 0.28) | 0.26  (0.14; 0.37) | 0.20  (0.08; 0.32) |
| **Barcelona Nou barris** |  |  |  |  |  |  |  |  |  |  |  |
| N | 239 |  | 17 | 11 | 11 | 32 | 16 | 44 | 44 | 35 | 49 |
| Rate  (95% CI) | 1.00  (0.99; 1.00) |  | 0.07  (-0.05; 0.19) | 0.05  (-0.08; 0.17) | 0.05  (-0.08; 0.17) | 0.13  (0.01; 0.25) | 0.07  (-0.06; 0.19) | 0.18  (0.07; 0.30) | 0.19  (0.07; 0.30) | 0.15  (0.03; 0.26) | 0.20  (0.09; 0.32) |
| **Barcelona Sant Andreu** |  |  |  |  |  |  |  |  |  |  |  |
| N | 293 |  | 21 | 23 | 11 | 20 | 42 | 36 | 39 | 57 | 53 |
| Rate  (95% CI) | 1.38  (1.24; 1.55) |  | 0.10  (0.01; 0.19) | 0.11  (-0.02; 0.24) | 0.05  (-0.08; 0.17) | 0.10  (-0.03; 0.23) | 0.20  (0.08; 0.32) | 0.17  (0.05; 0.30) | 0.19  (0.07; 0.31) | 0.27  (0.16; 0.39) | 0.25  (0.13; 0.37) |
| **Alt Penedès-Garraf** |  |  |  |  |  |  |  |  |  |  |  |
| N | 516 |  | 41 | 41 | 40 | 40 | 68 | 75 | 82 | 91 | 96 |
| Rate  (95% CI) | 1.15  (1.05; 1.25) |  | 0.10  (0.01; 0.19) | 0.09  (0.00; 0.18) | 0.09  (0.00; 0.18) | 0.09  (0.00; 0.18) | 0.15  (0.07; 0.24) | 0.17  (0.08; 0.25) | 0.18  (0.10; 0.27) | 0.20  (0.12; 0.28) | 0.21  (0.13; 0.30) |

**Table S1 (continuation). Autism spectrum disorders prevalence rate (%) in 2017 and ASD diagnosis incidence rate (%) between 2009 and 2017 among children between 2 to 17 years old by healthcare areas of the Catalonia region**

|  |  |  |  |  |  |  |  |  |  |  |  |
| --- | --- | --- | --- | --- | --- | --- | --- | --- | --- | --- | --- |
|  | **Prevalence** |  | **Incidence** | | | | | | | |  |
| **Healthcare area** | **2017** |  | **2009** | **2010** | **2011** | **2012** | **2013** | **2014** | **2015** | **2016** | **2017** |
| **Baix Llobregat Centre - Litoral i l'Hospitalet de Llobregat** |  |  |  |  |  |  |  |  |  |  |  |
| N | 2317 |  | 84 | 186 | 170 | 212 | 282 | 308 | 359 | 375 | 462 |
| Rate  (95% CI) | 1.47  (1.41; 1.53) |  | 0.05  (0.01; 0.10) | 0.12  (0.07; 0.17) | 0.11  (0.06; 0.15) | 0.13  (0.09; 0.18) | 0.18  (0.13; 0.22) | 0.19  (0.15; 0.24) | 0.23  (0.18; 0.27) | 0.24  (0.19; 0.28) | 0.29  (0.25; 0.33) |
| **Barcelonès Nord i Maresme** |  |  |  |  |  |  |  |  |  |  |  |
| N | 1654 |  | 80 | 135 | 144 | 165 | 231 | 257 | 271 | 258 | 299 |
| Rate  (95% CI) | 1.38  (1.31; 1.44) |  | 0.07  (0.01; 0.12) | 0.11  (0.06; 0.17) | 0.12  (0.07; 0.17) | 0.14  (0.08; 0.19) | 0.19  (0.14; 0.24) | 0.21  (0.16; 0.26) | 0.22  (0.17; 0.27) | 0.21  (0.16; 0.26) | 0.25  (0.20; 0.30) |
| CI: 95% confidence interval; N: number of children with ASD. | | | |  |  |  |  |  |  |  |  |

**Figure S1.**


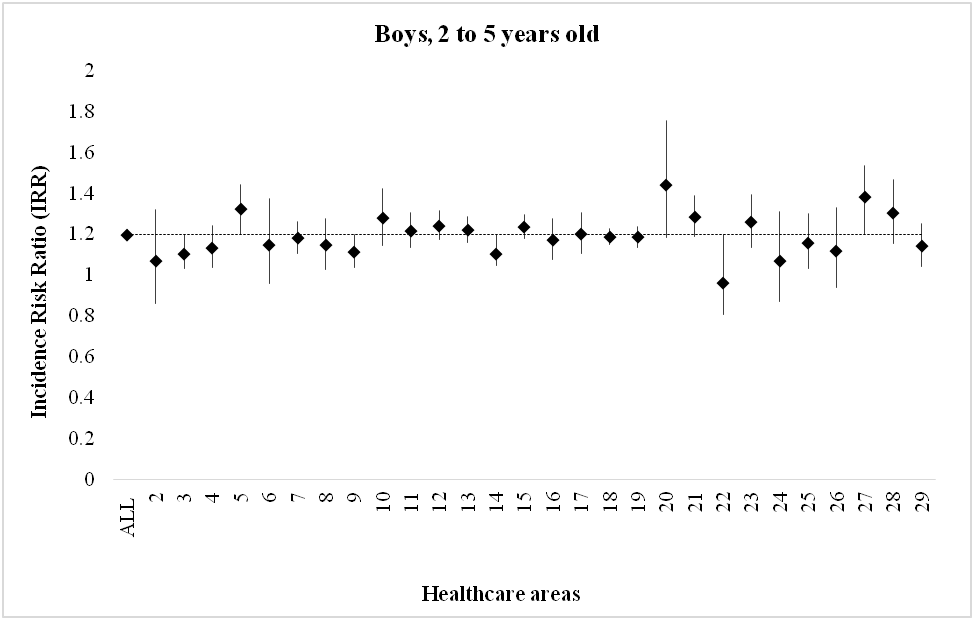
**A**

**B**


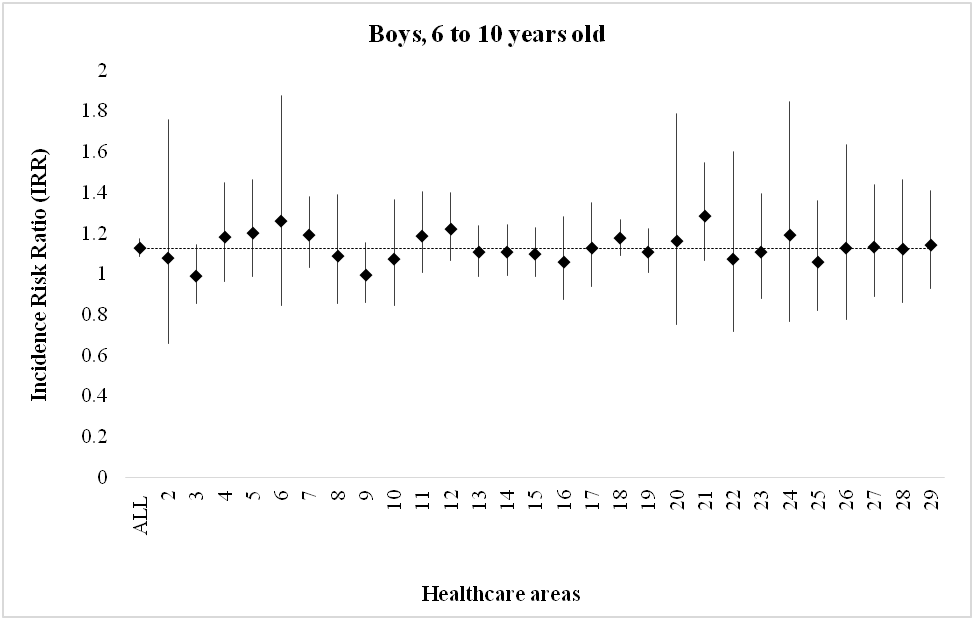


**C**


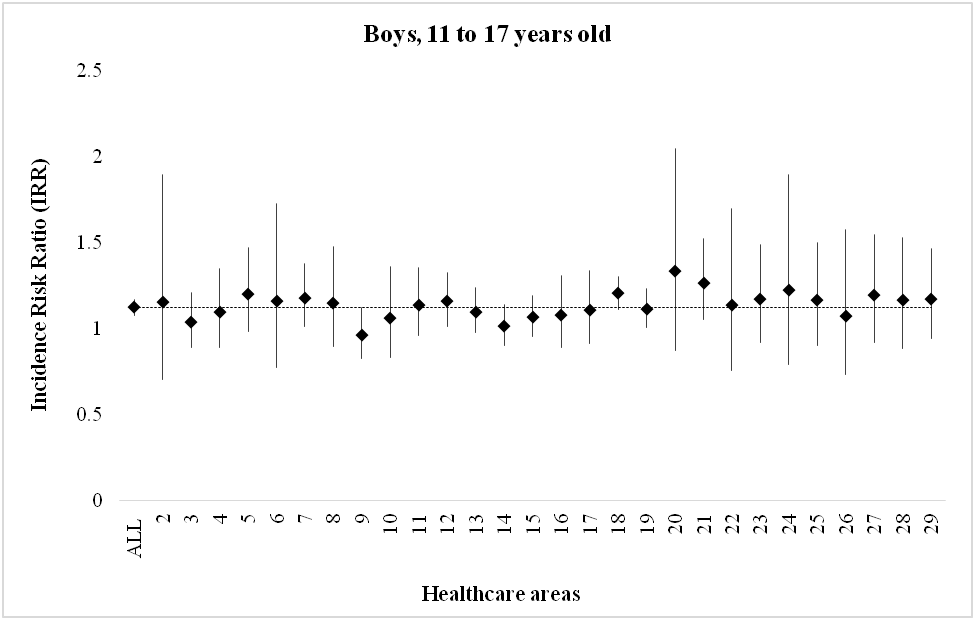


**D**


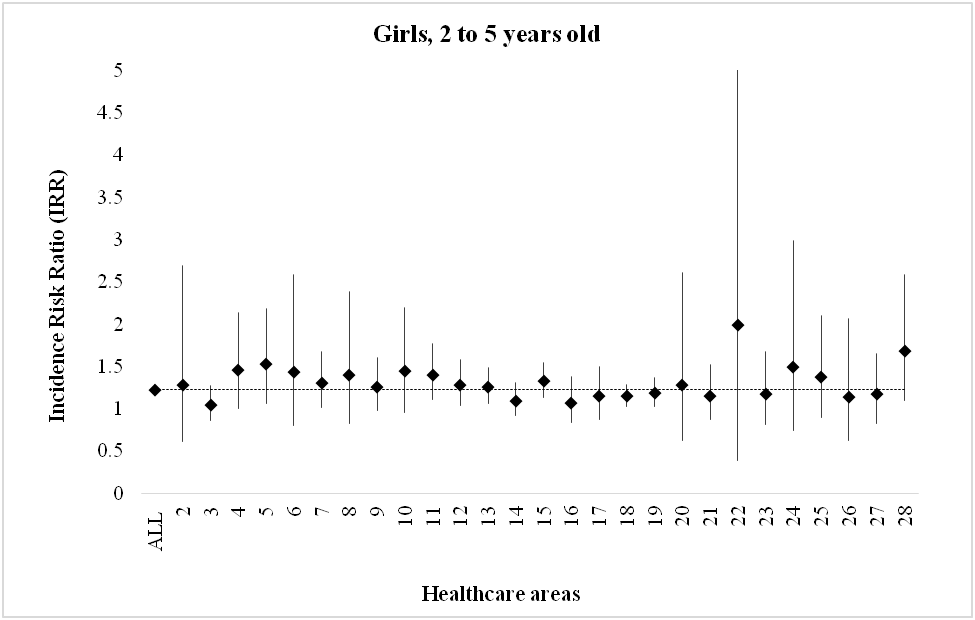


**E**


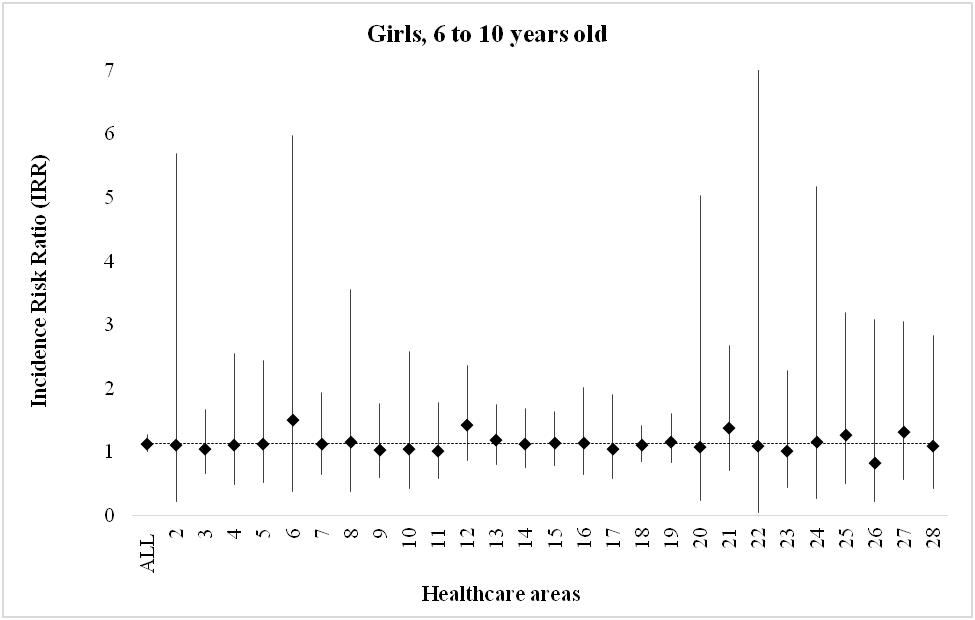


**F**

**
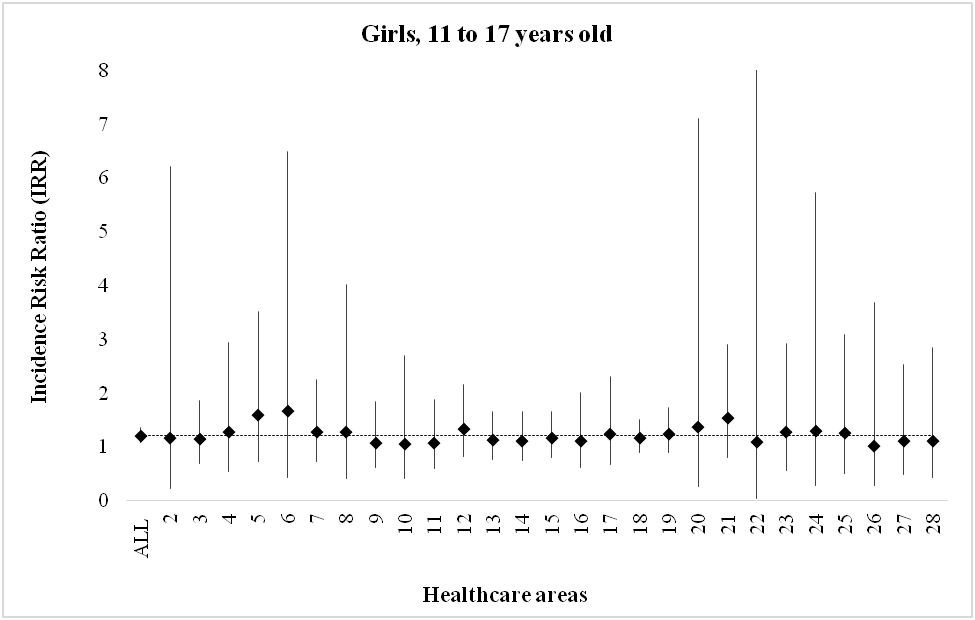
**
